# Invasive Infection With Purpureocillium lilacinum: A Case of Fungal Peritonitis

**DOI:** 10.7759/cureus.99526

**Published:** 2025-12-18

**Authors:** John M West, Lissete Whitaker, James Grubbs, Hasan Samra

**Affiliations:** 1 Division of Infectious Diseases, Medical College of Georgia, Augusta University, Augusta, USA; 2 Division of Pathology, Medical College of Georgia, Augusta University, Augusta, USA

**Keywords:** fungal peritonitis, isavuconazole, paecilomyces lilacinus, peritoneal dialysis (pd), purpureocillium lilacinum

## Abstract

*Purpureocillium lilacinum* is an environmental mold that is a rare yet emerging cause of opportunistic infection in humans. When infection does occur, it most often involves ocular or cutaneous structures, although other infections are increasingly reported. *P. lilacinum* has notably been documented to display resistance to several mainstay antifungal drugs. Here, we describe a case of peritoneal dialysis-associated mold peritonitis due to *P. lilacinum*. The patient was a 15-year-old female presenting with abdominal pain and a clogged peritoneal dialysis catheter. Peritoneal fluid cultures displayed mold growth, which would subsequently be identified as *P. lilacinum*. After initial treatment failure with fluconazole, the patient was successfully treated with a long course of isavuconazole. This case highlights just the fourth reported case of *P. lilacinum* peritonitis in the medical literature and the first treated with isavuconazole.

## Introduction

*Purpureocillium lilacinum*, formerly classified as *Paecilomyces lilacinus*, is a hyaline hyphomycete with a worldwide distribution that is often found in soil [1]. This species is known to cause various infections in both immunocompromised and immunocompetent hosts. As a human pathogen, it seemingly displays a tropism for ocular structures, with many of the reported cases, including keratitis and endophthalmitis, while cutaneous and subcutaneous infections represent the next largest group [2]. *P. lilacinum* has more rarely been documented to cause a variety of other infections, including reported cases of sinusitis, pulmonary infection, onychomycosis, vaginitis, endocarditis, and bursitis. Peritonitis can be a complication of peritoneal dialysis (PD) and is most often bacterial in etiology. PD-associated peritonitis is a rare manifestation of *P. lilacinum* infection, with a total of just two published cases in the medical literature to date, as well as one further unpublished case in the FungiScoperegistry [3-5]. Here, we report a case of PD-associated peritonitis treated successfully with isavuconazonium sulfate (prodrug of isavuconazole, brand name Cresemba).

## Case presentation

A 15-year-old female initially presented with one day of diffuse abdominal pain, cloudy PD fluid, and clogging of the PD catheter. Her past medical history was significant for end-stage renal disease on home PD for several years secondary to bilateral grade IV vesicoureteral reflux. The patient’s history additionally included a recent hospitalization approximately five months prior due to bacterial peritonitis with PD fluid cultures growing *Pantoea dispersa*, treated successfully with intraperitoneal (IP) cefepime and oral fluconazole.

Upon presentation to the emergency department, the patient’s vitals were notable for a blood pressure of 92/53 mmHg and tachycardia to a rate of 122 beats per minute. She was afebrile. Physical exam was remarkable for diffuse abdominal tenderness to palpation with white flecks noted within the lumen of the PD catheter. Initial laboratory results included a white blood cell count of 11.3 x 10^9^/L and C-reactive protein of 2.4 mg/dL (see Table 1).

**Table 1:** Initial laboratory findings AST: aspartate aminotransferase; ALT: alanine aminotransferase

Blood test	Value	Reference range
White blood cells (cells/mm^3^)	11,300	4,500-11,000
Hemoglobin (g/dL)	11.3	12.0-16.0
Albumin (g/dL)	3.6	3.2-4.8
Blood urea nitrogen (mg/dL)	26	9-23
Creatinine (mg/dL)	9.75	0.80-1.20
Sodium (mmol/L)	129	132-146
Potassium (mmol/L)	4.1	3.5-5.5
Chloride (mmol/L)	91	99-109
Calcium (mg/dL)	10.8	8.7-10.4
Glucose (mg/dL)	80	74-106
AST (U/L)	13	11-35
ALT (U/L)	12	10-49
C-reactive protein (mg/dL)	2.4	0-0.5
Peritoneal fluid analysis		
Total nucleated cells (cells/mm^3^)	3,688	-
Neutrophils (%)	53	-
Lymphocytes (%)	14	-
Monocytes (%)	28	-
Basophils (%)	4	-

Due to clinical suspicion for recurrent bacterial peritonitis, the patient received empiric intravenous (IV) cefepime 1 g every 24 hours and IV vancomycin 20 mg/kg. She was also started on IP cefepime and IP vancomycin upon admission. IV fluconazole was subsequently added at 6 mg/kg every 24 hours. After several days without growth on a blood culture obtained, systemic antibiotics were discontinued, with the patient continuing to receive IP cefepime and vancomycin, as well as IV fluconazole.

**Figure 1:**
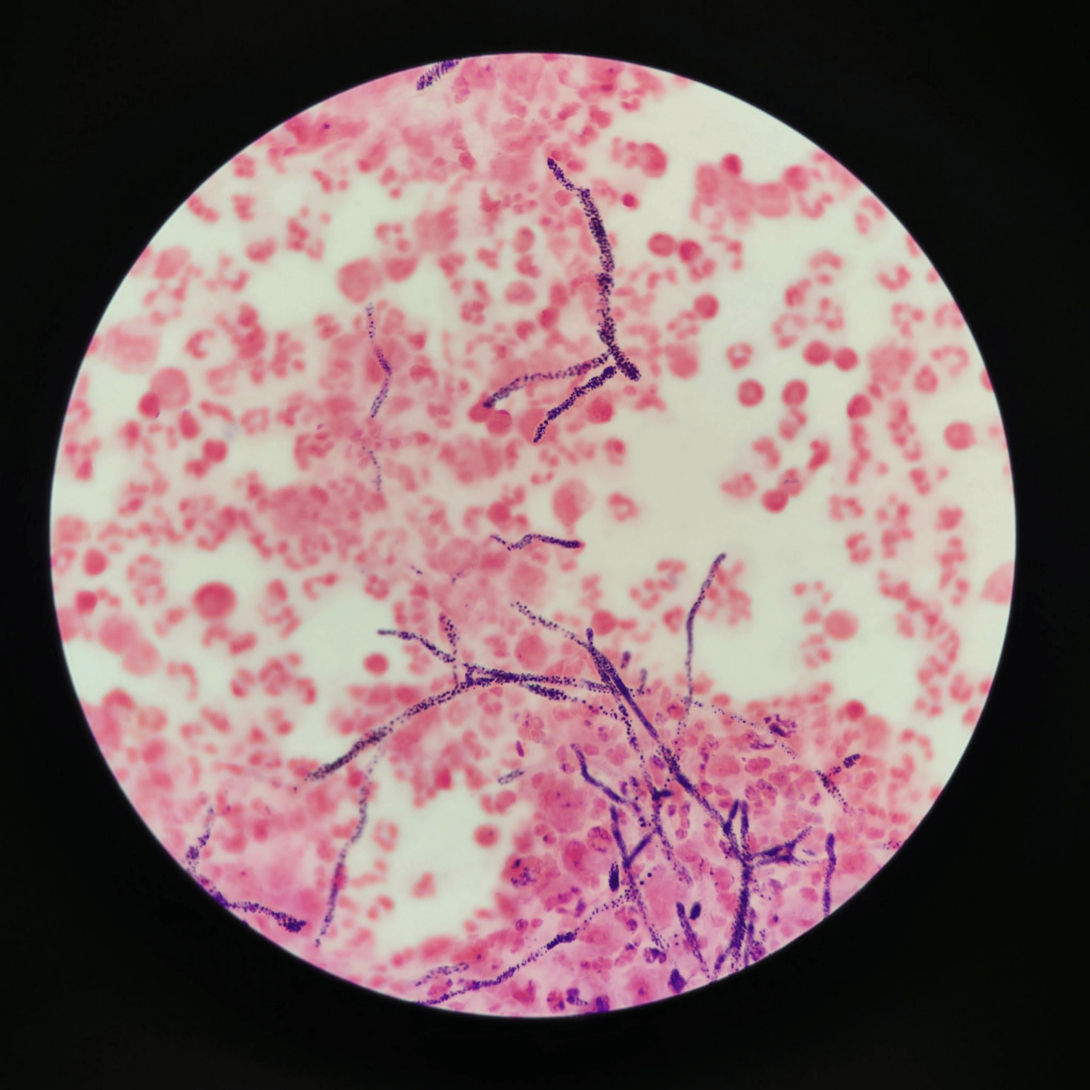
Gram-stained image of peritoneal fluid Small, non-septate hyphae with no yeast forms can be visualized with abundant neutrophils occupying much of the field.

The initial bacterial culture of the PD fluid displayed no bacterial growth but did show evidence of mold growth, requiring a send-out to the Mayo Clinic for genetic identification, ultimately coming back as *P. lilacinum* (see Figure 1). The total nucleated cell count was 3,688 cells/mm^3^ with 53% neutrophils, 28% basophils, and 14% lymphocytes (see Table 1).

After several days of the above antimicrobials, the patient had clinically improved significantly, and repeat cultures were no longer displaying mold growth. Removal of the PD catheter was discussed but deferred at that time due to symptomatic improvement. She was discharged home with a plan for continued IP cefepime and vancomycin as an outpatient and oral fluconazole to complete a total of 14 days of antifungal therapy.

Approximately 10 days after discharge, the patient presented to the emergency department once again with recurrent abdominal pain and nausea, for which she was readmitted to the hospital. A culture of peritoneal fluid obtained on hospital day two again displayed growth of *P. lilacinum*. A blood culture obtained upon presentation to the emergency department was without growth. At this time, the infectious diseases service was consulted. After initially receiving fluconazole, she was switched to oral isavuconazole with a loading dose of 372 mg every eight hours for the first six doses, followed by 372 mg daily thereafter, with plans for a longer treatment course given her recurrence. The PD catheter was additionally removed on hospital day four of the second hospitalization due to concern for colonization, with the patient transitioning to hemodialysis. She was again discharged on hospital day nine with resolution of her symptoms. She was to continue isavuconazole at home with outpatient follow-up.

At the time of follow-up, 15 weeks after the initial presentation, the patient continued to show clinical improvement. The C-reactive protein level had decreased to 0.577 mg/dL, showing a continued downtrend, and she reported minimal abdominal pain.

## Discussion

*P. lilacinum* is an extremely rare cause of peritonitis. Indeed, filamentous fungi in general are relatively rarely implicated in peritonitis, with the majority of fungal peritonitis cases being attributed to *Candida* species [6]. Among those caused by filamentous fungi, *Aspergillus* species and *Penicillium* species are more common [6]. However, invasive infections caused by filamentous fungi are becoming increasingly common and are often more difficult to treat [7]. Additionally, due to the limited number of reported cases of *P. lilacinum* peritonitis, there is exceedingly sparse data available on effective treatment modalities.

Antifungal susceptibility data for *P. lilacinum* are available from *in vitro* studies, which generally show resistance to many common antifungal agents. In particular, *P. lilacinum* has frequently been found to have high minimum inhibitory concentrations (MIC) for amphotericin B, suggesting an intrinsic resistance [8]. A study by Monpierre et al. similarly found high MICs for amphotericin B in all 70 of the clinical isolates of *P. lilacinum* they tested, as well as for echinocandins as a class [9]. Azoles displayed significantly better activity; while posaconazole was found to have the greatest activity, voriconazole and isavuconazole also showed good activity against *P. lilacinum*, with itraconazole having intermediate activity [9].

The first recorded case of PD-associated peritonitis caused by *P. lilacinum* in the literature was published by Chang et al. in 2008, wherein they documented a case of a 15-year-old male with a previous history of reflux nephropathy status post renal transplantation complicated by chronic rejection who was treated to good effect by three months of combination therapy of oral voriconazole and terbinafine [3]. The other case available in the literature, published by Wolley et al. in 2012, similarly reported successful treatment with 12 months of oral voriconazole and terbinafine [4].

Given the sparsity of available data surrounding treatment for *P. lilacinum* peritonitis, optimal treatment regimens have not been clearly established, both in terms of antimicrobial agent selection and length of therapy. Despite the limited data, guidelines for the treatment of rare molds do exist, including those published by the American Society for Microbiology, in collaboration with the European Confederation of Medical Mycology and the International Society for Human and Animal Mycology [10]. For *P. lilacinum* infection, these guidelines recommend first-line treatment with voriconazole, and in cases of cutaneous or subcutaneous infection, voriconazole and terbinafine [10]. However, it is important to note that these guidelines cite only a single case of PD-associated peritonitis and instead rely predominantly on cases of other, more frequent types of infection caused by *P. lilacinum*, which themselves still only include relatively few documented cases. In addition to displaying good activity in several *in vitro* studies, isavuconazole has been documented to effectively treat *P. lilacinum* clinically as well. Yang et al. recently reported a case of pulmonary *P. lilacinum* infection that was initially treated with oral voriconazole for three days but was subsequently discontinued in favor of isavuconazole due to significant adverse effects that were attributed to voriconazole use, including hallucinations and tremor [11]. They reported a good clinical response to isavuconazole therapy in that patient, with no significant adverse effects noted [11].

Voriconazole has indeed been noted to have significant drug interactions and a nontrivial side effect profile. In a randomized-controlled trial, isavuconazole was found to be non-inferior to voriconazole in the treatment of invasive mold disease while being associated with significantly fewer drug-related adverse effects [12]. Additionally, unlike voriconazole and other members of the class, such as posaconazole and itraconazole, isavuconazole does not display the same degree of pharmacokinetic variability between patients, which necessitates therapeutic drug monitoring in those drugs [13].

In the present case, we report an immunocompetent patient on PD with culture-proven *P. lilacinum* peritonitis, treated successfully with isavuconazole. This finding aligns with those reported by Yang et al. [11], as well as with available *in vitro* susceptibility data. Additionally, our patient was not noted to have developed any significant drug-related adverse effects.

Interestingly, our patient did improve clinically after being treated with fluconazole upon initially presenting to the hospital, although the infection relapsed fairly quickly. This does suggest some meaningful degree of activity by fluconazole; however, it remains unclear whether the initial treatment failure can be attributed to antifungal agent choice or inadequate length of therapy. It is also important to note that the PD catheter was not removed during the first hospitalization, and therefore source control was not obtained. Although our patient was cured following treatment with isavuconazole, the close proximity of this change in treatment regimen to the removal of the PD catheter makes it difficult to discern their relative contributions to the patient's cure.

Given the particular rarity of *P. lilacinum* peritonitis, more research is needed to better elucidate optimal treatment. In particular, future studies may aim to better ascertain the role of isavuconazole in treating *P. lilacinum* infection, given its success in treating patients such as ours and its significant pharmacologic advantages over voriconazole and posaconazole.

## Conclusions

PD-associated peritonitis caused by *P. lilacinum* is extraordinarily rare, with no clearly optimal treatment regimen, although the rare instances that have been documented have reported success with voriconazole-based regimens. Isavuconazole has also shown good activity against *P. lilacinum* in both *in vitro* studies and clinically. In our patient, isavuconazole was similarly found to be effective with no notable adverse effects. Isavuconazole represents a promising yet little-studied antifungal agent for the treatment of *P. lilacinum* infections; however, given the marked paucity of data available at present, significant further investigation is necessary.
